# The Financial Burden Associated With Conservatively Managed Epistaxis

**DOI:** 10.7759/cureus.49250

**Published:** 2023-11-22

**Authors:** Abubaker Elamin, Laith Sinan, Amena Al Saad, Ahmed Bayoumi, Abdelrahman Ezzat

**Affiliations:** 1 General Surgery, Humanitas University, Milan, ITA; 2 Otolaryngology, Nottingham University Hospitals, Nottingham, GBR; 3 Orthopedic Surgery, Nottingham University Hospitals, Nottingham, GBR; 4 Otolaryngology, United Lincolnshire Hospitals, Lincoln, GBR; 5 Otolaryngology - Head and Neck Surgery, United Lincolnshire Hospitals, Lincoln, GBR

**Keywords:** direct health cost, cost reduction, economy, conservative, epistaxis, financial burden

## Abstract

Aim: Each year, approximately 25,000 patients present to NHS hospitals in the UK with epistaxis. This study aims to investigate the financial implications of epistaxis, focusing on cases managed conservatively. Specifically, the research explores the average cost of hospital bed stays, the length of hospitalization, and the impact of blood thinners on healthcare expenses.

Methods: A retrospective study spanning June 2022 to June 2023 collected data from electronic health records at our local district general hospital. Patients meeting inclusion criteria were analyzed for demographic information, duration of hospital stay, use of blood thinners, and outcomes. Statistical software (SAS and Excel) was used for data analysis.

Results: Out of 126 patients, conservatively managed epistaxis cases (n = 119) had an average age of 73.9 years, with 53.6% males. The mortality rate was 4.5%. The average hospital stay was 2.92 days. Approximately 57% of patients were taking blood thinners. The average cost of a hospital bed stay for epistaxis patients was £1,712.84, with a £259.69 difference between those on blood thinners and those not.

Conclusion: Epistaxis imposes a significant financial burden on hospitals, with conservatively managed cases incurring substantial costs. Recognizing and addressing the financial implications of epistaxis is essential for healthcare providers and policymakers. Investment in preventative medicine and patient education can potentially reduce the incidence of epistaxis and alleviate the financial burden on healthcare systems.

## Introduction

Epistaxis or nosebleeds is a common condition that affects a significant percentage of the population. Its severity can range from being minor to a potentially life-threatening emergency [1]. The incidence of epistaxis is estimated to be around 60% in the general population, with up to 98% of patients with hereditary hemorrhagic telangiectasia (HHT) experiencing epistaxis during their lifetime [2]. Each year, approximately 25,000 patients present to NHS hospitals in the UK with epistaxis [3]. Epistaxis can be caused by several factors, including local and systemic causes. High blood pressure, trauma, and idiopathic factors are among the leading causes of epistaxis [4]. In patients with HHT, the pathogenesis of epistaxis is associated with abnormal angiogenesis and telangiectasia [2].

The anatomy of the nasal passages is important in understanding both anterior and posterior epistaxis because the location of bleeding is a key distinguishing factor between the two types.

Anterior epistaxis

Plexus: Anterior epistaxis, originates from the front part of the nasal passages (Kiesselbach's plexus or Little's area). This region is a complex vascular network located on the nasal septum [5]. 

Vessels: Kiesselbach's plexus is formed by the convergence of several blood vessels, including branches of the septal branch of the superior labial artery, anterior septal arteries, anterior ethmoid artery, and septal branch of the sphenopalatine artery. These blood vessels supply the nasal septum and adjacent mucous membranes [5].

Membrane: The nasal mucous membrane is a moist and delicate lining that helps filter and humidify the air we breathe. This mucous membrane can become vulnerable to damage from various factors, leading to bleeding in cases of anterior epistaxis [6].

Posterior epistaxis

Artery: Posterior epistaxis, originates from the blood vessels in the back part of the nasal passages. The sphenopalatine artery, a significant artery supplying the nasal cavity, is the main cause [7].

Vessels: The sphenopalatine artery and its branches are located deeper within the nasal cavity, making them less accessible and more challenging to manage when bleeding occurs. Other arteries, such as branches of the carotid arteries, can also be involved in posterior epistaxis [7].

Septum and nasal turbinates: The nasal turbinates and nasal septum are vital structures in the nasal passages. The septum is the central wall dividing the nose into left and right sides, while the turbinates are bony structures covered in mucous membranes that help warm and humidify inhaled air. Posterior epistaxis often involves bleeding from blood vessels located on or near these structures [8].

In some cases of posterior epistaxis, bleeding may extend into the nasopharynx, which is the upper part of the throat behind the nasal cavity. This can result in blood being swallowed or flowing down the throat, potentially causing other symptoms such as sore throat, or coughing up blood [9].

Anterior epistaxis is more common and often less severe, while on the other hand, posterior epistaxis can be more challenging to control due to the deeper location of the bleeding vessels. Differentiating between the two types of epistaxis is important for healthcare providers when determining the appropriate treatment approach. This is largely due to the fact that posterior epistaxis often requires more invasive methods to control the bleeding.

There are multiple treatment options available when managing epistaxis: conservative, medical, and surgical approaches can be employed depending on the severity and underlying cause of the condition [2]. Sphenopalatine artery surgeries, such as cauterization and ligation, have been found to be effective in cases of refractory idiopathic epistaxis [10]. Moreover, the Gelatin-thrombin matrix has been investigated as a first-line treatment for posterior epistaxis, showing promising results in a clinical trial [1]. Bevacizumab, an angiogenesis inhibitor, has also been explored as a potential treatment for HHT-related epistaxis, although further research is needed to establish its efficacy [11]. The prevalence and management of epistaxis can vary across different populations and healthcare settings. Studies conducted in Tanzania and Nigeria have reported the prevalence of epistaxis and the common causes in their respective regions [12,13]. Additionally, studies from India and Nigeria have highlighted the importance of conservative management as a preferred first-line treatment for epistaxis [14,15].

The management of epistaxis can involve various treatment options, including insertion of intranasal packs, nasal cautery, surgery, or interventional radiology [3]. These interventions often require hospital admission, leading to additional costs for the healthcare system. Healthcare expenditure represented around 11% of gross domestic product (GDP) in 2022, which is lower than the figure in 2021 of 12% [16]. This difference was caused by nominal expansion in healthcare expenditure being surpassed by the growth in the overall economy in 2022 [16].

The financial burden of epistaxis on hospitals in the NHS can be explained by multiple factors. Firstly, the cost of providing health care for patients with epistaxis can be significant. This includes the cost of healthcare professional services, medications, and procedures. Additionally, the length of hospital stay for patients with epistaxis contributes to the financial burden. It is estimated that the average cost of a general ward per bedday in the NHS is £586.59 [17]. Epistaxis can carry a substantial financial burden for patients and healthcare systems. The above-mentioned are direct cost but another significant contributor are the indirect costs associated with hospital admissions. These include, but are not limited to, lost jobs, time out of work for family members, and lost school days for kids.

This research aims to deduce the financial implications of epistaxis and evaluate the average cost for patients undergoing conservative management. Through a comprehensive analysis of medical expenses, resource utilization, and healthcare outcomes, this study sheds light on the economic aspects of epistaxis, offering valuable insights for patients, healthcare providers, and policymakers.

## Materials and methods

We conducted a retrospective study over a 12-month period, spanning from June 2022 to June 2023, with the aim of investigating the demographic characteristics, clinical variables, and outcomes of patients admitted with epistaxis. The study design involved the collection and analysis of data from electronic health records in Lincoln County Hospital (LCH). LCH is a district general hospital typical of the county, it is a 602-bed hospital including an ICU and is part of United Lincolnshire Hospitals NHS Trust. All patients admitted to the hospital during the study period with a primary diagnosis of epistaxis were included in the study. Patients who underwent surgical interventions for epistaxis were excluded from the analysis to focus exclusively on conservatively managed cases.

Patients meeting the following criteria were included in the study - confirmed diagnosis of epistaxis, underwent conservative management (e.g., nasal packing, medication) as the primary treatment approach, and availability of complete medical and financial records. The following information was collected for each eligible patient - demographic data - age, and gender. Duration of hospital stay - the length of time each patient spent in the hospital for the management of epistaxis. Use of blood thinners - information regarding whether patients were taking blood thinners, including the type of blood thinners (anticoagulants or antiplatelets), was recorded. Outcome assessment - patients' outcomes were categorized as either mortality during hospitalization or discharge from the hospital.

Data analysis was performed using statistical software (SPSS; IBM Corp., Armonk, NY). Descriptive statistics were employed to summarize demographic information, duration of stay, use of blood thinners, and outcomes. Continuous variables were presented as means with standard deviations (SD), while categorical variables were presented as frequencies and percentages.

## Results

**Figure 1:**
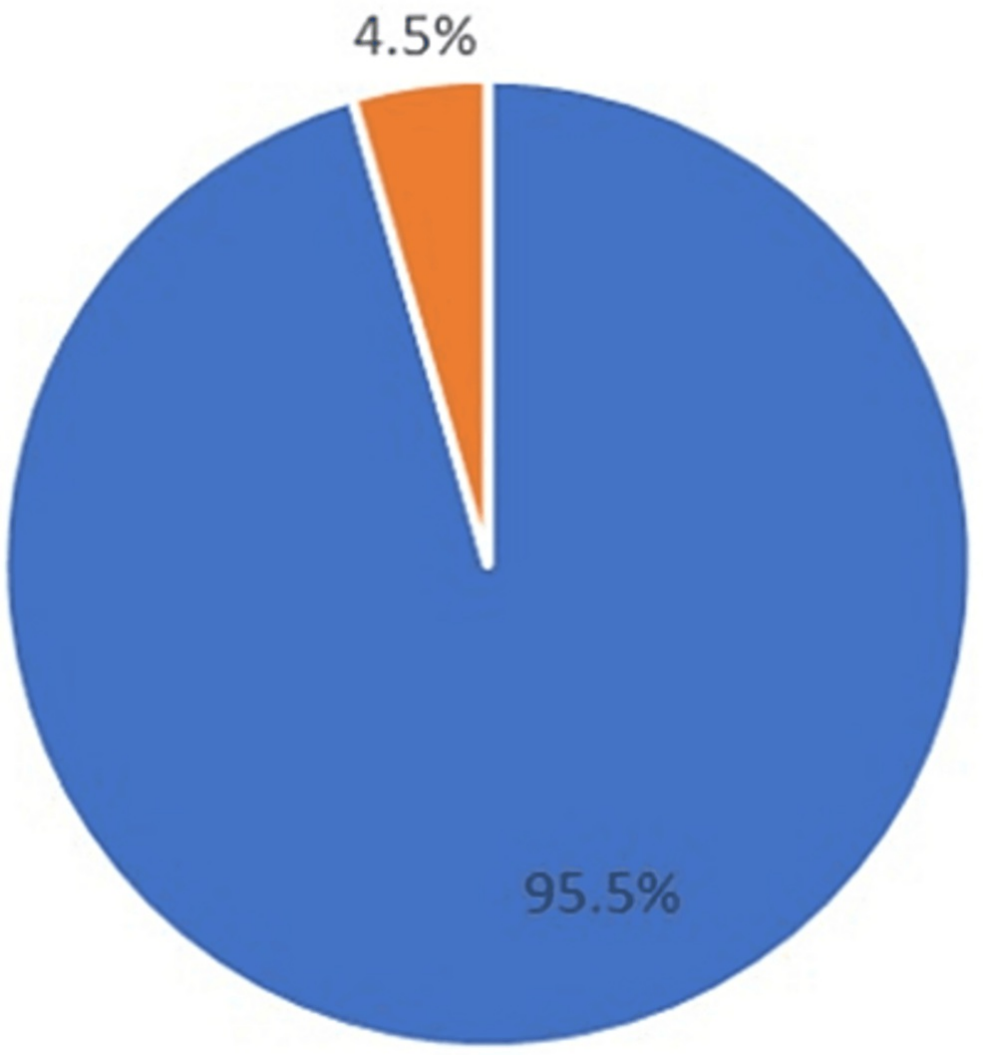
Mortality rate of hospitalized epistaxis Mortality (4.5%) represented as a percentage

A total of 126 patients with epistaxis were included in this study. Seven conservatively managed patients were excluded from the analysis due to the presence of comorbidities requiring extended hospital stays that were beyond the scope of this study. Another seven patients were excluded from this study as they were surgically managed epistaxis. The average age of conservatively managed patients was 73.9 years, with a median age of 78. Among the conservatively managed patients, 60 (53.6%) were male, and 52 (46.4%) were female. During the study period, the mortality rate was a total of 5 patients (4.5%) (Figure 1).

**Figure 2:**
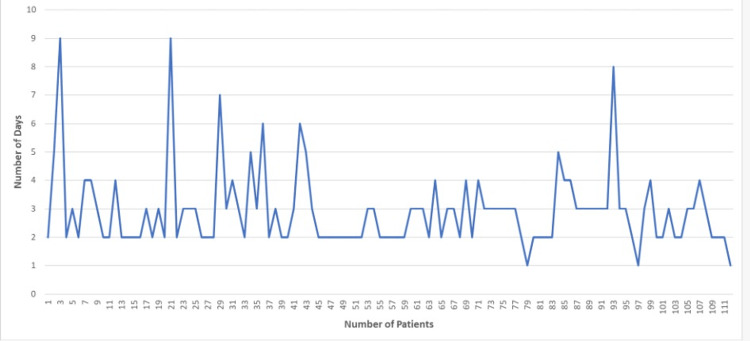
Length of hospital stay The data have been represented as N

The average length of hospital stay for conservatively managed patients with epistaxis was 2.92 days (Figure 2).

**Figure 3:**
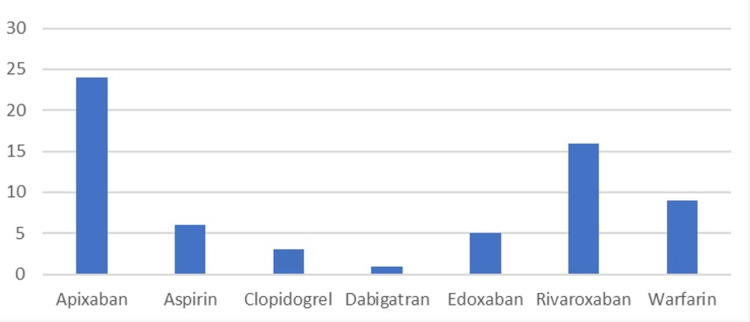
Antiplatelet/anticoagulant usage The data have been represented as N

Patients included in the study were on different antiplatelets and anticoagulants (Figure 3). Patients who were taking anticoagulants or antiplatelets had an average hospital stay of 3.11 days, while those not on blood thinners had an average stay of 2.67 days. The difference in the length of hospital stay between patients on anticoagulants/antiplatelets and those not taking them was 0.44 days.

Use of blood thinners - out of the conservatively managed patients, 64 (57%) were taking anticoagulants or antiplatelets, while 48 (43%) were not taking blood thinners. Financial burden - the average cost of a hospital bed stay for patients with epistaxis was found to be £1,712.84. Comparison of costs - the difference in the cost of hospital stays between patients who were taking blood thinners (anticoagulants or antiplatelets) and those who were not taking them was approximately £259.69.

## Discussion

While its clinical management is well-documented, the financial implications of epistaxis often remain understudied. This research endeavors to fill this knowledge gap by focusing on the financial burden associated with epistaxis, specifically in cases where conservative management is employed. Indirect costs, often underestimated, played a notable role in the overall financial burden associated with epistaxis. Patients frequently reported absenteeism from work, school, or daily activities, resulting in substantial indirect costs. Transportation expenses for medical visits and follow-up care also contribute to the financial burden.

Epistaxis, or nosebleeds, can impose a significant financial burden on hospitals. This study aimed to explore the financial implications of epistaxis on hospitals, shedding light on the costs associated with its management. By analyzing relevant data and considering the findings in the context of existing literature, this discussion section provides insights into the economic impact of epistaxis on healthcare systems. The findings of this study align with previous research on the financial burden of epistaxis. Villwock and Jones (2013) conducted a comparative study on epistaxis management in the United States and highlighted the various treatment modalities and associated costs [18]. Their study emphasized the role of hospitalization in the management of epistaxis, which can significantly contribute to the financial burden.

Similarly, our study focused on the costs incurred during hospital stays for patients with epistaxis. One of the key findings of our study was the average cost of a hospital bed stay for patients with epistaxis, which was found to be £1,712.84. This figure includes the direct costs of hospital beds and does not consider indirect costs. In addition, this cost provides us with an estimate of the financial burden and the cost can vary depending on factors such as the severity of the epistaxis, the length of hospital stays, and the specific hospital. Furthermore, our study shed light on the length of hospital stay for patients with epistaxis. The average length of stay for patients with epistaxis managed conservatively was found to be 2.92 days. This duration includes the time required for the management of epistaxis, including interventions such as medications and nasal packing. It is important to note that the length of hospital stay can impact the overall costs incurred by hospitals. Longer stays may lead to increased resource utilization and thus higher healthcare expenses.

Furthermore, another aspect explored in our study was the use of anticoagulants or antiplatelets among patients with epistaxis. Approximately 57% of conservatively managed patients were taking anticoagulants or antiplatelets. This finding is consistent with the literature, which highlights the association between the use of blood thinners and an increased risk of epistaxis. The presence of comorbidities and the need for continued anticoagulation therapy can further complicate the management of epistaxis and potentially contribute to the financial burden on hospitals. Considering the limitations of this study; firstly, it was conducted in a specific hospital setting and may not be representative of the financial burden experienced by all hospitals in the NHS.

Secondly, the study focused on the costs incurred during hospital stays and did not consider other aspects such as outpatient care or long-term follow-up costs. Future research could explore these areas to provide a more comprehensive understanding of the financial implications of epistaxis on hospitals. In conclusion, this study highlights the financial burden of epistaxis on hospitals. The average cost of a hospital bed stay for patients with epistaxis was found to be £1,712.84, with an average length of stay of 2.92 days. Many of the patients that required admission or prolonged hospital stays were those who were using blood thinners, this further adds to the complexity and cost of management. Our findings highlight the need for efficient resource allocation and cost-effective strategies in the management of epistaxis to reduce the financial burden on hospitals.

## Conclusions

Epistaxis poses a significant financial burden on hospitals. The average cost of hospital bed stays for patients with epistaxis, coupled with the length of hospital stay and the use of blood thinners, contributes to the overall economic impact. By recognizing and addressing the financial implications of epistaxis, healthcare providers and policymakers can work towards more efficient resource allocation, cost-effective care, and improved financial sustainability within healthcare systems. As noted, many of the causes of epistaxis are due to systemic illnesses such as hypertension. A potential solution to this would be further investment and emphasis on preventative medicine aimed at reversing or managing these conditions in primary care. Patient education and public health campaigns are pivotal tools that can be employed in order to reduce the incidence of these diseases and hence reduce the number of patients suffering from epistaxis.
